# Single cell sequencing analysis and transcriptome analysis constructed the liquid-liquid phase separation(LLPS)-related prognostic model for endometrial cancer

**DOI:** 10.3389/fonc.2022.1005472

**Published:** 2022-09-14

**Authors:** Jiayang Wang, Fei Meng, Fei Mao

**Affiliations:** ^1^ Department of Radiotherapy, The Affiliated Huaian No. 1 People’s Hospital of Nanjing Medical University, Huaian, China; ^2^ Department of Gynaecology, The Affiliated Huaian No. 1 People’s Hospital of Nanjing Medical University, Huaian, China; ^3^ Department of Urology, The Affiliated Huaian No. 1 People’s Hospital of Nanjing Medical University, Huaian, China

**Keywords:** liquid-liquid phase separation, endometrial cancer, immune microenvironment, single cell sequencing data, transcriptome data

## Abstract

**Background:**

Endometrial cancer is one of the most common gynecological tumors in developed countries. Our understanding of the pathogenesis of endometrial cancer and the changes in the immune microenvironment are still unclear. It is necessary to explore new biomarkers to guide the diagnosis and treatment of endometrial cancer.

**Methods:**

The GEO database was used to download the endometrial cancer single cell sequencing dataset GSE173682. The UCSC database was used to download transcriptome sequencing data. The validation set was the transcriptome dataset GSE119041, which was retrieved from the GEO database. On the DrLLPS website, liquid-liquid phase separation-related genes can be downloaded. Relevant hub genes were found using weighted co-expression network analysis and dimension reduction clustering analysis. Prognostic models were built using Lasso regression and univariate COX regression. Analyses of immune infiltration were employed to investigate the endometrial cancer immunological microenvironment. The expression of model genes in endometrial cancer was confirmed using a PCR test.

**Results:**

We created an LLPS-related predictive model for endometrial cancer by extensive study, and it consists of four genes: EIF2S2, SNRPC, PRELID1, and NDUFB9. Patients with endometrial cancer may be classified into high-risk and low-risk groups based on their risk scores, and those in the high-risk group had significantly worse prognoses (P<0.05). Additionally, there were notable variations in the immunological milieu between the groups at high and low risk. EIF2S2, SNRPC, PRELID1, and NDUFB9 were all up-regulated in endometrial cancer tissues, according to PCR results.

**Conclusions:**

Our study can provide a certain reference for the diagnosis and treatment of endometrial cancer.

## Introduction

The most common disease of the female reproductive system, endometrial cancer, is becoming more common everywhere (1). Both perimenopausal and postmenopausal women are impacted (2). It is difficult to diagnose and treat endometrial cancer because of its high incidence, which is thought to be mostly attributed to obesity (3). Bleeding is a common sign of endometrial cancer after menopause, which helps many people get diagnosed early (4). However, some people continue to experience occult symptoms, which delays diagnosis (5). Surgery is typically avoided because of the poor prognosis and limited therapy options for endometrial cancer in its advanced stages (6). Advanced endometrial cancer cannot be effectively treated with chemotherapy or radiotherapy (7). Therefore, the need for improved prognostic classification systems for endometrial cancer is critical.

Cancer growth-related signals and apoptosis resistance are both activated by a variety of events, including gene mutation, transcriptome alterations, epigenetic modification, and others (8). Liquid-liquid phase separation(LLPS) complicates the etiology of cancer (9). In the past, LLPS was believed to be a frequent occurrence and experimental principle in the fields of physics, chemistry, and pharmacy (10). The development of membraneless organelles and research led to the hypothesis that LLPS controls the synthesis of membraneless agglutines in healthy living cells, enabling a dynamic and steady reaction (11). LLPS may participate in a variety of signal transduction pathways, epigenetic control of cancer, and cancer genesis (12). On the other hand, it is yet unclear how LLPS affects endometrial cancer. The importance of LLPS in endometrial cancer has to be examined. It’s time to examine LLPS’s connection to endometrial cancer.

In this study, we studied the involvement of LLPS-related genes in endometrial cancer by single-cell sequencing analysis and transcriptome sequencing analysis, and created a predictive model to estimate the prognosis and immunological status of patients with endometrial cancer. Overall, our study presents novel therapeutic concepts and new biomarkers for endometrial cancer.

## Methods

### Single cell sequencing data download and processing

GEO (Gene Expression Omnbius) database (https://www.ncbi.nlm.nih.gov/geo/) provides many the transcriptome data and single cell sequencing data of the disease. From this database, the single cell sequencing dataset GSE173682 for endometrial cancer was retrieved. The dataset comprised both endometrial and ovarian cancer, and only five endometrial cancer samples were preserved for later analysis. The quality control method was as follows: 1) Genes expressed in less than three cells were deleted. 2) Cells with gene expression between 200 and 3000 were maintained. 3) Cells that retain fewer than 10 percent of mitochondrial genes. Sample and pericellular batch effects were eliminated and samples were combined using the “SCT” approach. The DIMS was set at 1:25, and the TSNE method was utilized to minimize the dimension of samples. K. Peram was 20, random seed was 2021, and all cells were grouped using KNN’s approach. Cells were annotated using SingleR’s approach.

### Transcriptome data downloading and processing

Data from many databases, including TCGA and TARGERT, are included in the UCSC database (https://xena.ucsc.edu/). GDC TCGA Endometrioid Cancer transcriptome data and clinical information were retrieved *via* the database. Through matching, a total of 533 samples with expression matrices and clinical data were found. The downloaded transcriptome data is HTseQ-FPKM. From the GEO database, the endometrial cancer dataset GSE119041 was taken, and it served as an independent external validation cohort. After log2 transformation, transcriptome data were used for future analysis.

### Download of LLPS related genes

A significant amount of pertinent LLPS phenotypic correlation gene is sent to the DrLLPS website (http://llps.biocuckoo.cn/). This database’s list of LLPS-related genes, which includes 3611 genes in total, can be downloaded by choosing the download module.

### Single-sample gene set enrichment analysis

Single-sample gene-set enrichment analysis is an extension of the GSEA method, which can compute and obtain enrichment scores for each sample and gene set pairing, showing the degree to which members of a given gene set in the sample are coordinately up-or down-regulated. In this study, we eventually computed and acquired the enrichment fraction of LLPS phenotype in each sample by this analysis method.

### Weighted gene co-expression network analysis

Co-expressed genes can be categorized into modules using WGCNA, and the relationship between modules and phenotype can be investigated. This approach was utilized in this investigation to identify genes associated with the LLPS phenotype. The pickSoftThreshold function of the R package “WGCNA” is used to find the best soft field value. Step sizes of 1:10 and 12:20 are set to 1 and 2, respectively. Set deepSplit to 2 and the minimum number of module genes to 100.

### Construction and validation of the prognostic model

First, univariate COX regression was used to identify the genes associated with prognosis. After that, the prognosis-related genes were further examined using LASSO regression, with the family set to “Cox” and Maxit set to 1000. The survival differences between the model’s high and low risk groups were investigated in the training cohort and validation cohort, as well as whether the model could more accurately classify patients’ risk categories.

### Immune infiltration analysis

Immunedeconv is a R package that is used on the Timer2.0 website (http://timer.comp-genomics.org/) to provide a more accurate measurement of immune infiltration. The findings of seven different calculation techniques were retrieved from the Timer2 website for each endometrial cancer sample. The variations in immune infiltration between the high and low risk groups in the model were investigated, and the results were displayed using a heat map.

### The construction of a nomogram

In order to more correctly assess the prognosis of patients, clinical data and sample models were combined using the R program “Regplot” and then presented as a nomogram.

### PCR was used to verify the expression of model genes

The 8 EC patients were chosen between October 2021 and July 2022, and their EC tissue as well as healthy uterine tissue was obtained for mRNA quantification and qRT-PCR testing. This study was approved by the Ethics Committee of Huai ‘an First People’s Hospital (No.KY-2022-084-01). Total cellular RNAs were isolated from cells using Trizol Reagent per the manufacturer’s instructions (Invitrogen, Carlsbad, CA, USA). Using the Takara reverse transcription kit, reverse transcription was completed (Otsu, Shiga, Japan). The QuantiTect SYBR Green PCR Kit and QuantStudio 1 for the real-time polymerase chain reaction were given by Thermo, Waltham, Massachusetts (RT-PCR). Relative quantification was determined using the -2^ΔΔCt^ method. The relative expression level of each gene’s messenger RNA (mRNA) was changed to match the mRNA for the enzyme glyceraldehyde-3-phosphate dehydrogenase (GAPDH). The primer sequence used was as follows: GAPDH: Forward primer: GAATGGGCAGCCGTTAGGAA; Reverse primer: CCCAATACGACCAAATCAGAGA. EIF2S2: Forward primer: AGGTGTAAAGATTGAAAGTGATGTT; Reverse primer: TGTTGTCTTCATCTTCTAGAGCTTC. SNRPC: Forward primer: ACTGCAGTGGAAGGAAACACA; Reverse primer: TGTTGAAATGCAGCCGTTGT. PRELID1:Forward primer: GTCTCCAGAGCTGTCCAGGAAT; Reverse primer: TGTCTCAACAAGTGTTTTGGAAGG. NDUFB9:Forward primer: GTAATGGCGTTCTTGGCGTC; Reverse primer: TTCAAACCGGGCTCTGGAC.

### Statistical analysis

Genes linked to prognosis were screened using a univariate COX analysis. The KM survival analysis was used to evaluate the results for the patients. To compare gene expression between high- and low-risk groups, the wlicox test was employed. Statistics were judged significant at P <0.05.

## Results

### Single cell sequencing data analysis

**Figure 1:**
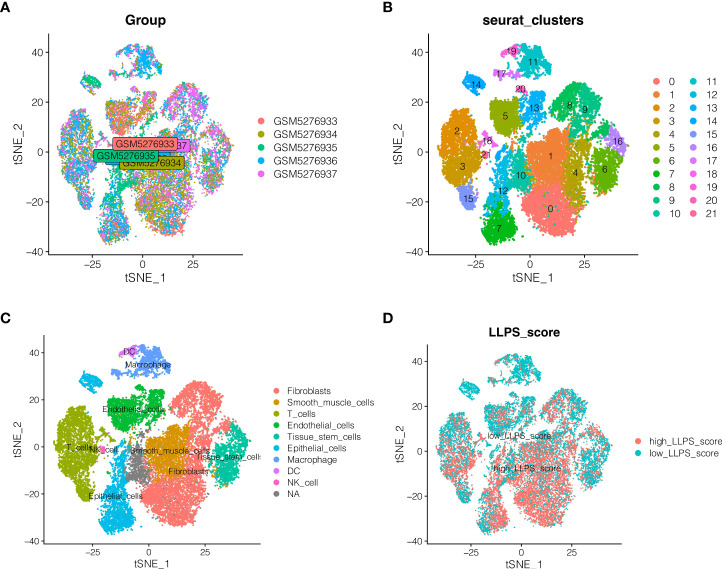
Single cell sequencing data analysis. **(A)** Detection of batch effects. **(B)** Dimension reduction and clustering. **(C)** \]Cell annotation. Nine cell types were annotated as Fibroblasts, Smoth muscle cells, T cells, epithelial cells, endothelial_cells, Tissue stem cells, macrophage, DC, And NK cells. **(D)** Distribution of LLPS score in different cells.

Single-cell sequencing data for endometrial cancer were studied. As indicated in **Figure 1A**, no significant batch effect was seen in the 5 endometrial cancer samples, which were appropriate for additional study. As shown in **Figure 1B**, all cells were clustered into 22 groups following dimension reduction and clustering. The cells were then further annotated, as shown in **Figure 1C**, where nine cell types were annotated as fibroblasts, smoth muscle cells, T cells, epithelial cells, endothelial_cells, tissue stem cells, macrophage, DC, And NK cells. The percentage of LLPS phenotype in each cell is determined according to the “PercentageFeatureSet” function of Seurat R package, and then separated into high LLPS score group and low LLPS score group according to the median value. As demonstrated in **Figure 1D**, high LLPS score group is largely distributed in fibroblasts and smoth muscle cells and epithelial cells group. And then differentially expressed genes analysis between the two groups were performed, by setting the conditions as | avg logFC | > 1 and the rectified adj p value < 0.05. A total of 2512 differentially expressed genes associated to LLPS in endometrial cancer were found.

### WGCNA analysis

**Figure 2:**
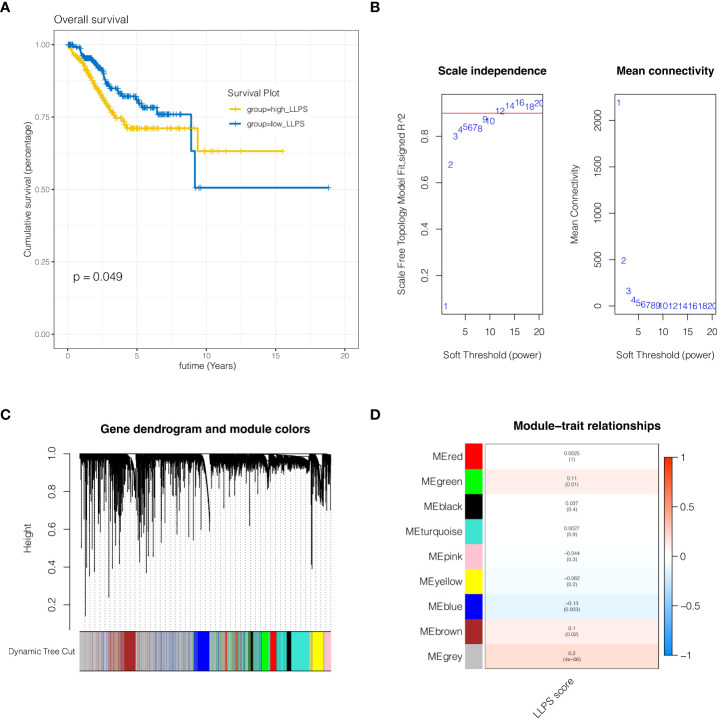
WGCNA analysis. **(A)** KM survival analysis. The survival prognosis of the high LLPS group was poor. **(B)** When the soft domain value is set to 9, it is discovered that R^2>0.8, and the data correspond to the power-law distribution, which is suitable for following analysis. **(C, D)** Clustering of modules. A total of 8 non-grey modules were identified, among which the green module had the strongest correlation with LLPS.

Genes associated to LLPS phenotype were further examined at the transcriptome level of endometrial cancer. Firstly, the enrichment fraction of LLPS phenotype in each sample was estimated by ssGSEA algorithm, and then separated into high LLPS group and low LLPS group according to the median value. As shown in **Figure 2A**, KM survival analysis suggested that the survival prognosis of the high LLPS group was poor. As shown in **Figure 2B**, when the soft domain value is set to 9, it is discovered that R^2>0.8, and the data correspond to the power-law distribution, which is suitable for following analysis. Moreover, with the increase in soft domain value, Mean Connectivity tends to stay steady. As indicated in **Figure 2C**, all genes were clustered into 8 non-grey modules, among which the green module exhibited the highest connection with LLPS score (**Figure 2D**, P <0.05). Then 1004 genes in the green module were selected out.

### Construction and validation of the prognostic model

**Figure 3:**
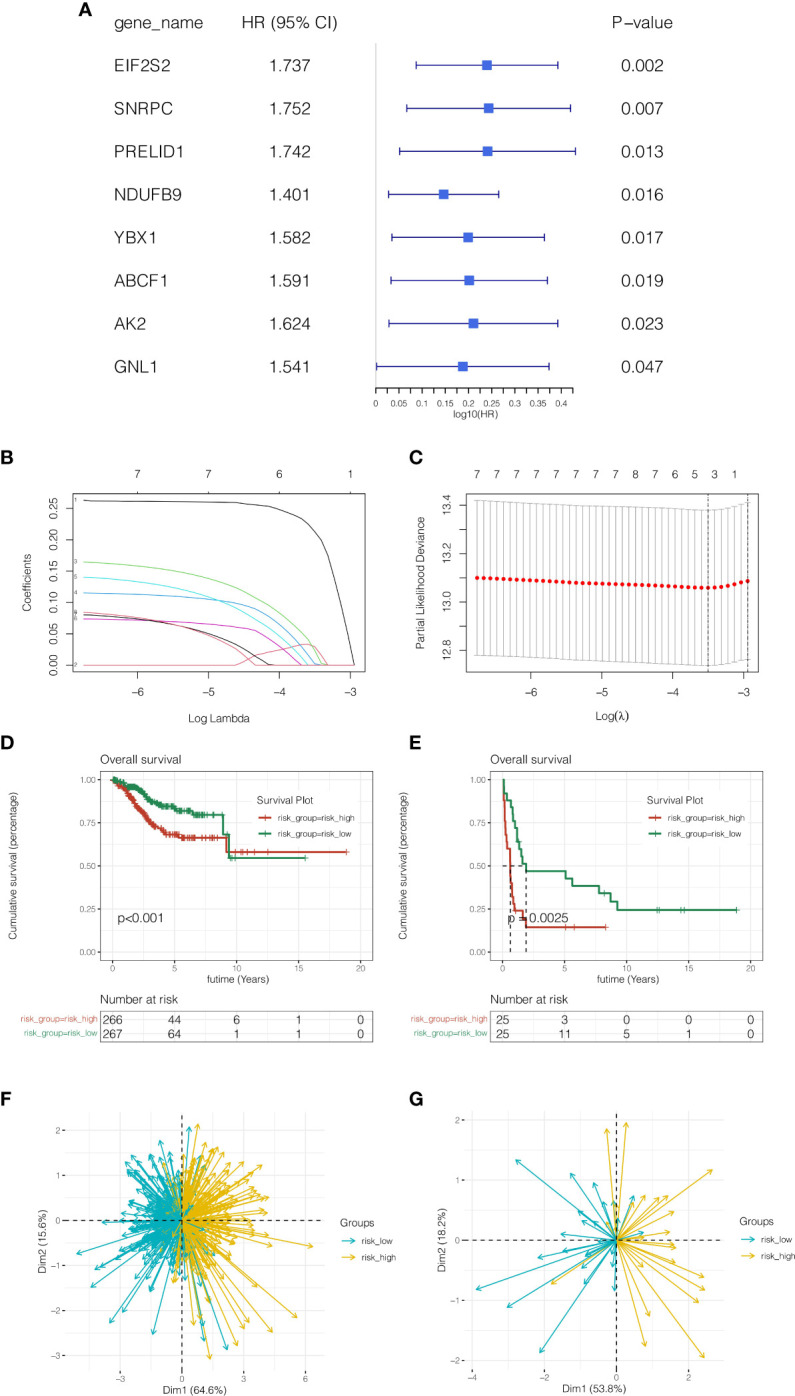
Construction and validation of the prognostic model. **(A)** Univariate COX analysis. **(B, C)** LASSO regression, four genes were included in the model, namely EIF2S2, SNRPC, PRELID1, and NDUFB9. **(D, E)** In both the training cohort TCGA and the external validation cohort GSE119041, it was observed that the prognosis of the risk high group was poorer than that of the risk low group (P <0.05). **(F, G)** Principal component analysis. This model can discriminate endometrial cancer patients well in both the training and validation cohorts.

To further identify LLPS-related genes in endometrial cancer at the single-cell level and tissue transcriptome sequencing level, 2512 genes obtained by the above single-cell sequencing analysis were intersected with 1004 genes of the green module analyzed by WGCNA, and a total of 158 genes were obtained. In order to further discover genes linked to prognosis, univariate COX analysis was done with P <0.05, and 8 genes related to prognosis in TCGA cohort and GSE119041 dataset were screened. As indicated in **Figure 3A**, these genes were EIF2S2, SNRPC, PRELID1, NDUFB9, YBX1, ABCF1, AK2 and GNL1. As shown in **Figures 3B, C**, when the best lambda value was 0.03 using LASSO regression, four genes were included in the model, namely EIF2S2, SNRPC, PRELID1, and NDUFB9. The calculation formula of the model was: riskScore = EIF2S2*(0.213) + SNRPC *(0.030) + PRELID1 *(0.024) + NDUFB9 *(0.002). (0.002). Then patients were separated into risk high group and risk low group according to the median riskscore value. As shown in **Figures 3D, E**, in both the training cohort TCGA and the external validation cohort GSE119041, it was observed that the prognosis of the risk high group was poorer than that of the risk low group (P <0.05). As shown in **Figures 3F, G**, this model can discriminate endometrial cancer patients well in both the training and validation cohorts.

### Unsupervised clustering analysis

**Figure 4:**
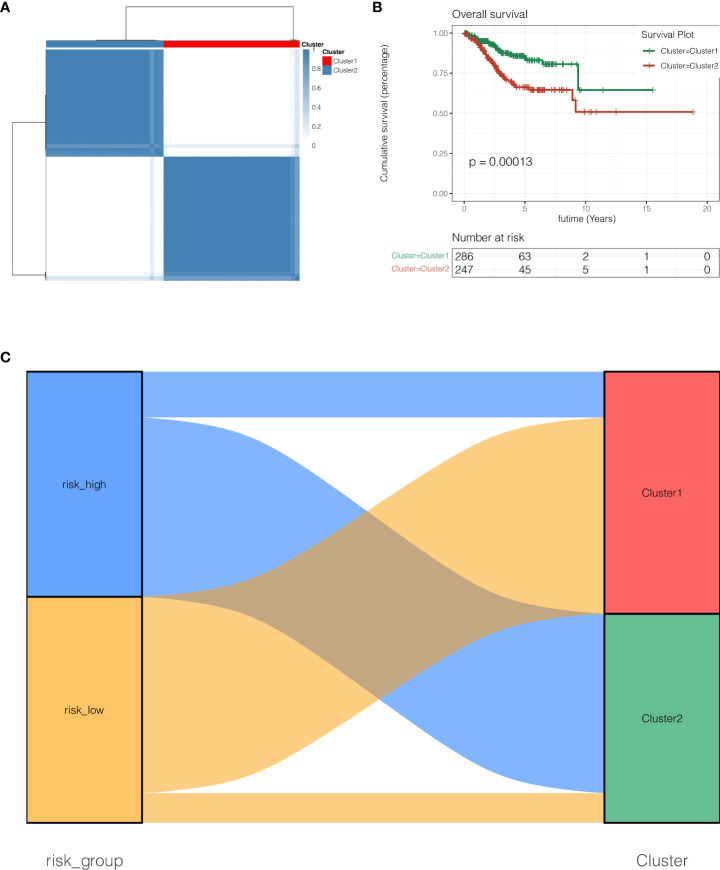
Unsupervised clustering analysis. **(A)** Unsupervised clustering clutered all endometrial cancer patients with two subgroups, Cluster1 and Cluster2. **(B)** the prognosis of Cluster2 was poorer than that of Cluster1 (P <0.001). **(C)** Risk high group mostly belongs to Cluster2, and risk low group mainly corresponds to Cluster1.

In TCGA cohort, as shown in **Figure 4A**, according to the expressions of EIF2S2, SNRPC, PRELID1 and NDUFB9, the R package “ConsensusClusterPlus” was used, clusterAlg was set as “PAM”, Distance was set as “Euclidean”, the random seed was set as 123456 and unsupervised clustering was done. All endometrial cancer patients were diagnosed with two subgroups, Cluster1 and Cluster2. As demonstrated in **Figure 4B**, the prognosis of Cluster2 was poorer than that of Cluster1 (P <0.001). As indicated in **Figure 4C**, risk high group mostly belongs to Cluster2, and risk low group mainly corresponds to Cluster1.

### Expression of model genes in cell subtypes

**Figure 5:**
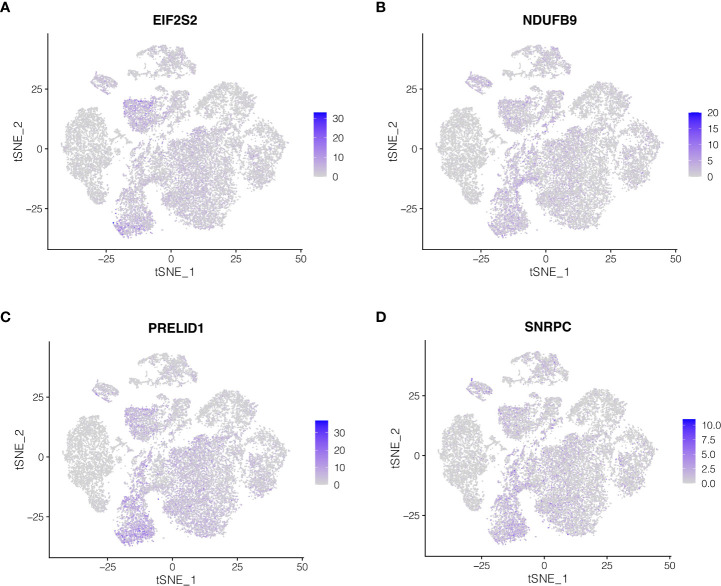
**(A-D)** Expression of model genes in cell subtypes.

The expression of the four genes in the model was then studied at the single-cell level. As indicated in **Figures 5A–D**, EIF2S2 and PRELID1 genes are largely expressed in epithelial cells and endothelial cells, while NDUFB9 and SNRPC genes are mainly expressed in epithelial cells, endothelial cells, fibroblasts and smoth muscle cells.

### Immune infiltration analysis

**Figure 6:**
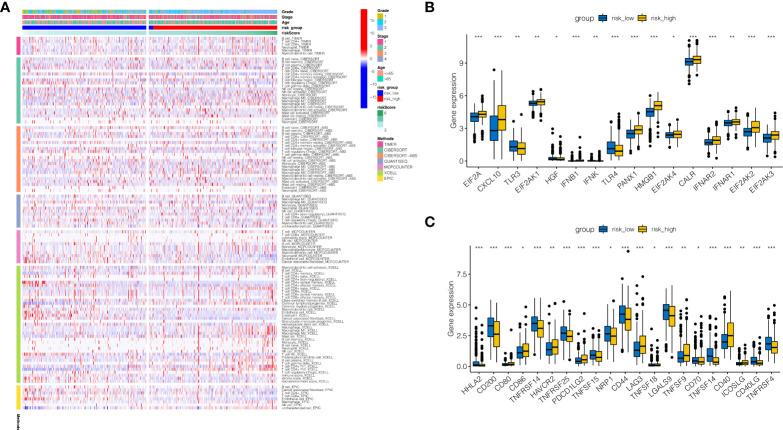
Immune infiltration analysis. **(A)** Immune infiltration landscape in high-risk and low-risk groups. **(B)** Tumor necrosis factor(TNF)-related genes were generally significantly expressed in the risk high group, such as EIF2A, CXCL10, and TLR3. **(C)** Immune checkpoint associated genes CD80, CD86, HAVCR2, PDCD1LG2, LAG3, TNFSF9, CD40, and ICOSLG were strongly expressed in RISK HIGH group. HHLA2, CD200, TNFRSF14, TNFRSF25, TNFSF15, NRP1, CD44, LGALS9, CD70, TNFSF14, CD40LG, and TNFRSF4 tended to be strongly expressed in risk low group. *P < 0.05, **P < 0.01, ***P < 0.001.

To further study the reasons for the difference in prognosis between risk high and risk low groups, correlation analysis of immune infiltration was done. As indicated in **Figure 6A**, macrophage was found to be relatively infiltrated in the risk high group, whereas T cell was largely infiltrated in the risk low group. As indicated in **Figure 6B**, tumor necrosis factor(TNF)-related genes were generally significantly expressed in the risk high group, such as EIF2A, CXCL10, and TLR3. As demonstrated in **Figure 6C**, immune checkpoint associated genes CD80, CD86, HAVCR2, PDCD1LG2, LAG3, TNFSF9, CD40, and ICOSLG were strongly expressed in RISK HIGH group. HHLA2, CD200, TNFRSF14, TNFRSF25, TNFSF15, NRP1, CD44, LGALS9, CD70, TNFSF14, CD40LG, and TNFRSF4 tended to be strongly expressed in risk low group.

### The construction of a nomogram

**Figure 7:**
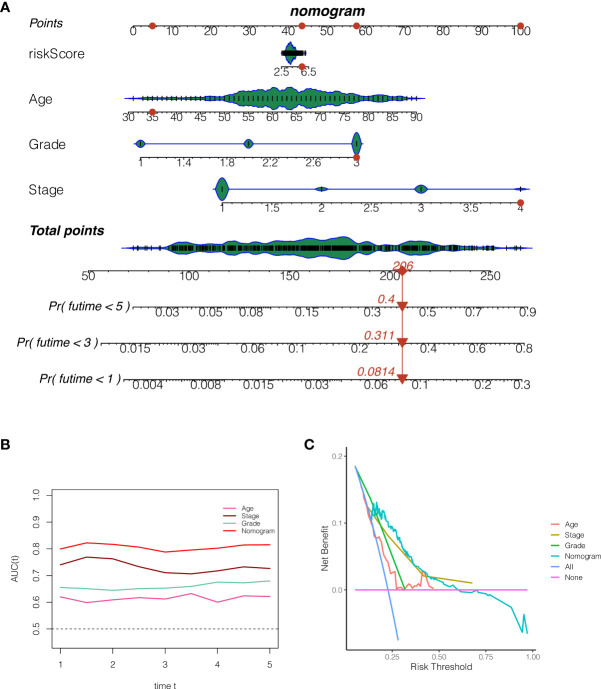
The construction of a nomogram. **(A)** The 1, 3, and 5-year death rates of TCGA-BS-A0TE patients were 0.0814, 0.3110, and 0.4000, respectively. **(B)** Continuous prognostic ROC analysis showed that the AUC of nomogram in determining the prognosis of patients was steady at around 0.8, which was better than other clinical indicators, such as age, stage and grade. **(C)** Decision curve analysis.

In order to properly evaluate the prognosis of patients, the clinical data of patients and the risk score of the model were merged. As shown in **Figure 7A**, the 1, 3, and 5-year death rates of TCGA-BS-A0TE patients were 0.0814, 0.3110, and 0.4000, respectively. As shown in **Figure 7B**, continuous prognostic ROC analysis showed that the AUC of nomogram in determining the prognosis of patients was steady at around 0.8, which was better than other clinical indicators, such as age, stage and grade. As shown in **Figure 7C**, decision curve analysis reveals that patients who make timely therapeutic decisions according to nomogram will benefit more than age, stage and grade.

### PCR was used to verify the expression of model genes

**Figure 8:**
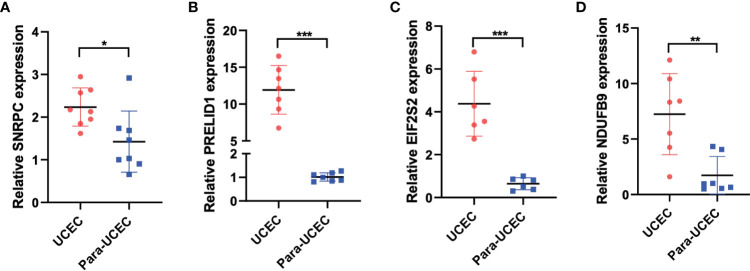
PCR was performed to verify the expression of model genes. **(A-D)** SNRPC, PRELID1, EIF2S2, and NDUFB9 were up-regulated in endometrial carcinoma. *p < 0.05; **p < 0.01; ***p < 0.001.

**Figure 8** displays the PCR findings. SNRPC, PRELID1, EIF2S2, and NDUFB9, four of the model’s genes, were discovered to have higher levels of expression in endometrial cancer than in nearby tissues (**Figures 8A–D**, *P<0.05, **P<0.01, ***P<0.001).

## Discussion

Endometrial cancer is the fourth most prevalent malignancy worldwide and the most common type of gynecologic cancer harming women’s health in affluent countries (13). Although most endometrial cancer patients are detected in the early stage because of vaginal bleeding, some individuals are still recognized late, typically accompanied by recurrence and metastasis, and the prognosis is dismal (14–16). For advanced, metastatic endometrial cancer, therapeutic choices are limited (17). Although developing targeted treatments and immunotherapies have been originally employed in the treatment of advanced endometrial cancer, low drug response and drug resistance are widespread, and many mechanisms of start and progression are still unexplained (18). It is of vital significance to research novel risk assessment methodologies and propose new biomarkers for endometrial cancer. Liquid-liquid phase separation (LLPS) was formerly thought to be involved in the development of membraneless intracellular organelles, and in recent years LLPS have been regarded to be highly relevant for many benign and malignant disorders (19–21). However, the significance of LLPS in endometrial cancer is not fully recognized. This study intends to explore the impact of LLPS-related genes in endometrial cancer.

In this investigation, a multiomics analysis was utilized to determine the expression, heterogeneity, prognostic value, and immunological evaluation value of LLPS-related genes in endometrial cancer. In order to explore the heterogeneity and activation status of LLPS in endometrial cancer, we first categorized endometrial cancer cells into several clusters using single-cell sequencing analysis. Based on this, we scored these cells and separated them into high-LLPS score groups and low-LLPS score groups. Genes that were differentially expressed between the two groups were also discovered. The heterogeneity of LLPS in endometrial cancer may be strongly correlated with these genes. The genes in this module were then believed to be strongly related with LLPS regulation in endometrial cancer after weighted co-expression network analysis (WGCNA) discovered green module that were highly associated with LLPS in endometrial cancer. By crossing these genes with differentially expressed genes previously discovered by single-cell sequencing research, these genes were recognized as LLPS hub genes in endometrial cancer. These genes served as the foundation for a predictive model that was built using COX regression and Lasso regression and included EIF2S2, SNRPC, PRELID1, and NDUFB9. Endometrial cancer patients were split into high-risk and low-risk groups using this predictive model, with the high-risk group having a noticeably worse prognosis. Unsupervised cluster analysis provides additional evidence of the model’s correctness. Between the high-risk group and the low-risk group, there were substantial differences in the amounts of immune cell infiltration and immune checkpoint gene expression, which may be a contributing factor to the different prognoses between the two groups and serve as a guide for immunotherapy.

According to certain studies, the four model genes EIF2S2, SNRPC, PRELID1, and NDUFB9 have significant roles in the development of cancer. The EIF2S2-LINC01600-MYC axis has been linked to the development and progression of tumors, according to Zhang et al (22). SNRPC, according to Zhang et al., can accelerate the development of hepatocellular carcinoma by triggering the epithelial-mesenchymal transition (23). Gillen and colleagues discovered that PRELID1’s alternative polyadenylation controls mitochondrial ROS signaling and the development of cancer (24). According to Li et al., down-regulation of NDUFB9 caused breast cancer cells to proliferate and spread by modulating mitochondrial metabolism (25). Our study reveals the role of these genes in endometrial cancer and provides a reference for understanding the role of these genes in regulating LLPS.

The single-cell dataset used in our study, GSE173682, was published in 2021. In that original study, the authors constructed a single-cell landscape of human gynecologic tumors using single-cell sequencing technology, revealing powerful heterogeneity in human gynecologic tumors (26). On this basis, we further analyzed the sequencing data and further explored the heterogeneity of LLPS in endometrial cancer, which provides a powerful reference for us to understand the pathogenesis and regulatory elements of endometrial cancer.

Currently, several bioinformatics signatures have been constructed in other tumors (27, 28). Qiu et al. constructed the signature of 11 LLPS-related genes in ovarian cancer by bioinformatics analysis, and accurately stratified the prognosis of ovarian cancer patients, among which patients in the high-risk group had significantly worse prognosis (29). And they found that LLPS-related genes were involved in several cancer-related pathways, such as MAPK signaling, cell cycle and DNA replication. Fang et al. constructed a signature composed of six LLPS-related genes in hepatocellular carcinoma by weighted co-expression network analysis and regression analysis to evaluate the prognosis and immune status of patients (30). Compared with these published studies, our study incorporated analysis of single-cell sequencing data and validated our conclusions using PCR assays with clinical samples. Our study can provide a reference for the diagnosis and treatment of endometrial cancer.

## Conclusions

We constructed a novel LLPS-related signature in endometrial cancer to assess patient prognosis and immune status. However, we lack corresponding clinical cohort to evaluate the practicality and accuracy of this signature. We will improve it in the future.

## Data availability statement

The original contributions presented in the study are included in the article/supplementary material. Further inquiries can be directed to the corresponding author.

## Ethics statement

The studies involving human participants were reviewed and approved by Ethics Committee of Huai ‘an First People’s Hospital. The patients/participants provided their written informed consent to participate in this study.

## Author contributions

JW designed the study. JW and FMe were involved in database search and statistical analyses. JW, FMe and FMa were involved in the writing of manuscript and its critical revision. All authors were responsible for the submission of the final version of the paper. All authors approved the final version. All authors agree to be accountable for all aspects of the work.

## Acknowledgments

We are very grateful for data provided by databases such as TCGA, GEO.

## Conflict of interest

The authors declare that the research was conducted in the absence of any commercial or financial relationships that could be construed as a potential conflict of interest.

## Publisher’s note

All claims expressed in this article are solely those of the authors and do not necessarily represent those of their affiliated organizations, or those of the publisher, the editors and the reviewers. Any product that may be evaluated in this article, or claim that may be made by its manufacturer, is not guaranteed or endorsed by the publisher.
